# Impacts of exercise, renin–angiotensin system modulation or both on skeletal muscle circadian gene expression

**DOI:** 10.1113/EP092318

**Published:** 2026-01-11

**Authors:** Emily L. Zumbro, Liliana C. Baptista, Taylor Taylor, Abbi R. Hernandez, Anisha Banerjee, Yi Sun, YouFeng Yang, Qiuhong Li, Thomas W. Buford

**Affiliations:** ^1^ Division of Gerontology, Geriatrics, and Palliative Care, Department of Medicine University of Alabama at Birmingham Birmingham Alabama USA; ^2^ Faculty of Sports Sciences and Physical Education University of Coimbra Coimbra Portugal; ^3^ Research Center in Sport and Physical Activity University of Coimbra Coimbra Portugal; ^4^ Department of Family and Community Medicine University of Alabama at Birmingham Birmingham Alabama USA; ^5^ Department of Life, Health, and Physical Sciences Gordon College Wenham Massachusetts USA; ^6^ Department of Ophthalmology, College of Medicine University of Florida Gainesville Florida USA; ^7^ Birmingham/Atlanta VA Geriatric Research Education and Clinical Center Birmingham VA Medical Center Birmingham Alabama USA

**Keywords:** circadian rhythm, exercise, probiotic, skeletal muscle

## Abstract

Ageing negatively affects quality of life and healthspan, and interventions are needed to slow this progressive decline. Previously, we have demonstrated the potential functional benefits of combining a genetically modified probiotic (GMP) targeting the non‐canonical arm of the renin–angiotensin system (RAS) with exercise training. Initial RNAseq studies indicated the potential of the interventions to influence circadian physiology. Therefore, the objective of this study was to evaluate the expression of circadian‐related genes in the tibialis anterior and soleus muscles in male and female aged rats in response to the administration of the GMP, exercise training and multiple controls. Following 12 weeks of the intervention, circadian‐related genes were differentially expressed in male and female aged rats and between tissues, primarily influenced by the exercise intervention, with potential additive effects of the GMP. Several genes were also significantly associated with measures of physical performance. Thus, combining exercise with a RAS‐related GMP may have potential functional benefits in late life, potentially related to circadian‐related impacts within skeletal muscle.

## Abstract (highlights)

- **What is the central question of this study?**
  How does an Ang (1–7)‐expressing genetically modified probiotic in combination with exercise training impact skeletal muscle circadian rhythm in older rats?
- **What is the main finding and its importance?**
  Although exercise is a primary factor in altering circadian rhythm gene expression, the probiotic may have an additional influence on circadian rhythm genes. This is important as such ageing negatively impacts circadian rhythms, and this study provides interventions that can be utilised to combat these age‐associated changes.

## 1 INTRODUCTION

Physical and cognitive decline is prominent in adults aged 65 and older in the United States (CDC, 2021) as a result of a number of factors, including, but not limited to, changes in the cardiovascular system and the loss of muscle mass and strength (Bisset & Howlett, 2019; Panza et al., 2018). With the advancement of medicine and the subsequent increase in life expectancy, physical and cognitive decline continue to impact older adults. Regular physical activity is beneficial for attenuating physical (Lozano‐Montoya et al., 2017; Vlietstra et al., 2018) and cognitive decline (Biazus‐Sehn et al., 2020; Karssemeijer et al., 2017; Paillard, 2015) whilst also preserving muscle quality (Chambers et al., 2020; Zeng et al., 2020) and promoting cardiovascular health (Chen et al., 2022; El Assar et al., 2022). However, mode and intensity of exercise, along with other inter‐individual factors, impact physiological responses to training (Chung et al., 2021; Gonzales et al., 2022; Harman & Martín, 2020; Ramnath et al., 2018; Sampaio et al., 2020). Furthermore, a heterogeneous response to exercise training in older adults influenced by several factors (i.e., medications, inflammatory status, sex hormones, oxidative stress response, disease/chronic condition status, sleeping patterns, epigenetics and circadian rhythm) is evident and is a current research focus (Erickson et al., 2023). Due to the multi‐component complexity of ageing and the exercise training response, more efficient treatment strategies are needed to address this heterogeneous response and provide alternative methods for those at risk of physical and cognitive decline but who are unable to partake in regular exercise training.

The renin–angiotensin system (RAS) is of interest due to its effects on tissues and organs outside of the circulation that influence physical and cognitive health (Basso et al., 2005; Carter & Groban, 2008; Zhou et al., 2015). Historically, the focus on the RAS has been the canonical arm of blood pressure regulation via the vasoconstrictive properties of angiotensin II (Ang II). However, a newer focus on the RAS involves the non‐canonical arm, which is represented by the peptide angiotensin (1–7) [Ang(1–7)]. Ang(1–7) acts by binding to the Mas receptor and promoting vasodilation, angiogenesis, anti‐inflammation and reduced lipid deposition (Iusuf et al., 2008; Lelis et al., 2019; Rodrigues Prestes et al., 2017; Santos, 2014). Angiotensin‐converting enzyme 2 (ACE2) converts both angiotensin I and angiotensin II into Ang(1–7), increasing the potential of the positive effects of the non‐canonical arm (Passos‐Silva et al., 2013). Additionally, Ang(1–7) is known to have beneficial effects on skeletal muscle, including improved muscle size, lipid content and insulin sensitivity (Aguirre et al., 2020; Aravena et al., 2020; Rivera et al., 2020; Valero‐Breton et al., 2023; Williams et al., 2016; Yamamoto et al., 2020).

The half‐life of Ang(1–7) is short, and therefore novel therapeutic approaches are needed to capitalise upon its positive benefits. We previously reported the efficacy of a genetically modified probiotic (GMP) for increasing circulating concentrations of Ang(1–7), whilst also providing other benefits relevant to gut, brain and physical health both in isolation and in combination with exercise (Baptista et al., 2023; Buford et al., 2020; Carter et al., 2019; Hernandez et al., 2022). In this prior work, we also conducted an extensive multi‐tissue analysis from RNA sequencing (RNAseq) to explore the underlying mechanisms of the beneficial health effects of the Ang(1–7)‐expressing GMP, exercise training and the combination of the two. Amongst the strongest signals from these RNAseq experiments was a potential impact on the expression of genes related to the regulation of circadian rhythms in skeletal muscle.

Circadian clocks are present in virtually all cells, and their role is to support homeostasis through regulation of a daily programme of gene expression (Cederroth et al., 2019). The circadian clock mechanism is defined by a self‐sustaining transcription–translation feedback loop (TTFL) that underlies the daily rhythms in behaviour and physiology (Partch et al., 2014). The core clock in mammals includes the two basic helix–loop–helix PAS transcription factors *CLOCK* and *BMAL1* that function as the positive limb. *CLOCK* and *BMAL1* regulate expression of the negative limb genes, *PER1/2* and *CRY1/2*. The PER and *CRY* proteins will subsequently feed back to inhibit *CLOCK:BMAL1* transcriptional activity. An auxiliary loop that supports the TTFL involves the retinoid‐related orphan receptors (*ROR*) and *Rev‐ERBs* (*NR1D1/2*). The *RORs* function to enhance *BMAL1* gene expression, whilst *NR1D1/2* will inhibit the expression of *BMAL1*. Therefore, the mechanisms regulating rhythmic physiology are complex, and studies have shown that disruption to the clock mechanism leads, in general, to poorer outcomes such that in any deviation from normal rhythmicity may have deleterious effects. Although rats are nocturnal, with activity and feeding largely in the dark phase, the clock mechanism in the skeletal muscle is similar to that in diurnal humans based on rest:activity patterns (Gutierrez‐Monreal et al., 2020; Harfmann et al., 2015).

It is now recognised that circadian clocks exist in skeletal muscle, and several labs have shown that altered muscle clocks are associated with insulin resistance and changes in substrate metabolism (Dyar et al., 2014; Harfmann et al., 2015; Gabriel et al., 2021). The muscle clocks can be modulated by exercise or muscle contractions, as they have been shown to function as a time cue for the muscle independent of the central clocks in the brain (Kemler et al., 2020; Wolff & Esser, 2012). However, the role acute and chronic exercise plays in regulating skeletal muscle circadian rhythms in older adults and between sexes is unclear (Bruns et al., 2020; de Souza Teixeira et al., 2020). Alternatively, the gut microbiome is suspected to influence host central and peripheral circadian rhythms, but scientific evidence is lacking (Cheng et al., 2022; Matenchuk et al., 2020; Parkar et al., 2019). Additionally, with the link of the RAS, blood pressure and circadian rhythm, our novel Ang(1–7)‐expressing GMP may influence muscle‐specific circadian rhythms (Ayyar & Sukumaran, 2021; Hastings et al., 2018; Ohashi et al., 2017; Wessel et al., 2007).

Therefore, based on the original RNAseq findings, the purpose of this project was to examine the differences between groups of circadian clock genes in male and female rats within tibialis anterior and soleus muscle, a type II and type I fibre‐prominent muscle, respectively, following an exercise‐training and Ang(1–7)‐expressing GMP administration protocol over the course of 12 weeks. Secondary analyses were conducted to examine the relationship of gene expression with performance measures (i.e., time to exhaustion and grip strength) in male and female aged rats and within muscle types.

## 2 METHODS

### 2.1 Ethical approval

All animal experimental procedures were approved and conducted in accordance with the guidelines set by the Institutional Animal Care and Use Committee (IACUC) at the University of Alabama at Birmingham (UAB; IACUC‐21360) and laws governed by the Public Health Service, Office of Laboratory Animal Welfare, United States Department of Agriculture and the American Association for Accreditation of Laboratory Animal Care.

### 2.2 Experimental design

The experimental procedures for this study have been described previously (Baptista et al., 2023; Buford et al., 2020; Carter et al., 2019; Hernandez et al., 2022). Briefly, male (*n* = 82) and female (*n* = 84) Fisher 344 × Brown Norway rats aged 24 months from the National Institute on Ageing colony were utilised in this 12‐week trial. Rats were randomly assigned to oral treatment groups [control: CON; probiotic: LP; and Ang(1–7)‐expressing probiotic: LPA] and further divided into exercise status groups (sedentary: Sed; exercise: Ex), six groups in total. All rats were fed a standard rodent chow (18% fat, no sucrose, 3.1 kcal/g; Harlan Teklad, Madison, WI, USA) and water available ad libitum. Of all rats randomly assigned, 63 males and 72 females survived and completed the intervention, and their tissues were harvested (Table 1). As a note, the COVID‐19 epidemic impacted tissue harvesting during the study, and thus, tissue was not collected from all rodents due to enforced social distancing protocols and staff availability.

**TABLE 1:** Distribution of rats per group for males and females and tissue availability for biochemical analyses.

	Male			Female		
Group	*n*	Tibialis anterior	Soleus	*n*	Tibialis anterior	Soleus
CON Sed	12	12	8	13	11	13
CON Ex	12	10	7	11	11	10
LP Sed	11	10	6	11	10	11
LP Ex	11	11	10	12	12	8
LPA Sed	9	9	8	12	9	12
LPA Ex	8	8	8	13	11	7

Abbreviations: CON Ex, control + exercise group; CON Sed, control + sedentary group; LP Ex, probiotic + exercise group; LP Sed, probiotic + sedentary group; LPA Ex, Ang(1–7)‐expressing probiotic + exercise group; LPA Sed, Ang(1–7)‐expressing probiotic + sedentary group.

Prior to the intervention, a treadmill acclimatization protocol was administered over the course of 2 weeks for all rats, as previously described (Baptista et al., 2023; Hernandez et al., 2022). All rodents were individually housed in an accredited facility at UAB under standard conditions of a 12 h light/12 h dark cycle (06.00–18.00 h) at 20–23°C, Zeitgeber Time 0 (ZT0). In other words, the lights were ON at 06.00 h. Oral gavages were administered to all oral treatment groups at 2 × 10^11^ colony‐forming units/kg body weight for the LP and LPA groups or an equal volume of buffer for the CON group 3×/week at 08.00 h (ZT2) following the acclimatization period. The development of the Ang(1–7)‐expressing GMP formulation has been described elsewhere (Buford et al., 2020). Briefly, the GMP were cultured at 37°C for 18 h in MRS (deMan Rogosa Sharpe) broth (BD Difco, Houston, TX, USA) with 5 µg/ml erythromycin, and bacteria were harvested by centrifugation at 5000 *g* for 20 min, then resuspended in phosphate‐buffered saline (PBS) for oral gavage. Prior to conducting experiments, colony counting was conducted to confirm optimal numbers of surviving bacteria. The volume range throughout the study for the oral gavage was 0.4–1.1 mL per dose.

**FIGURE 1:**
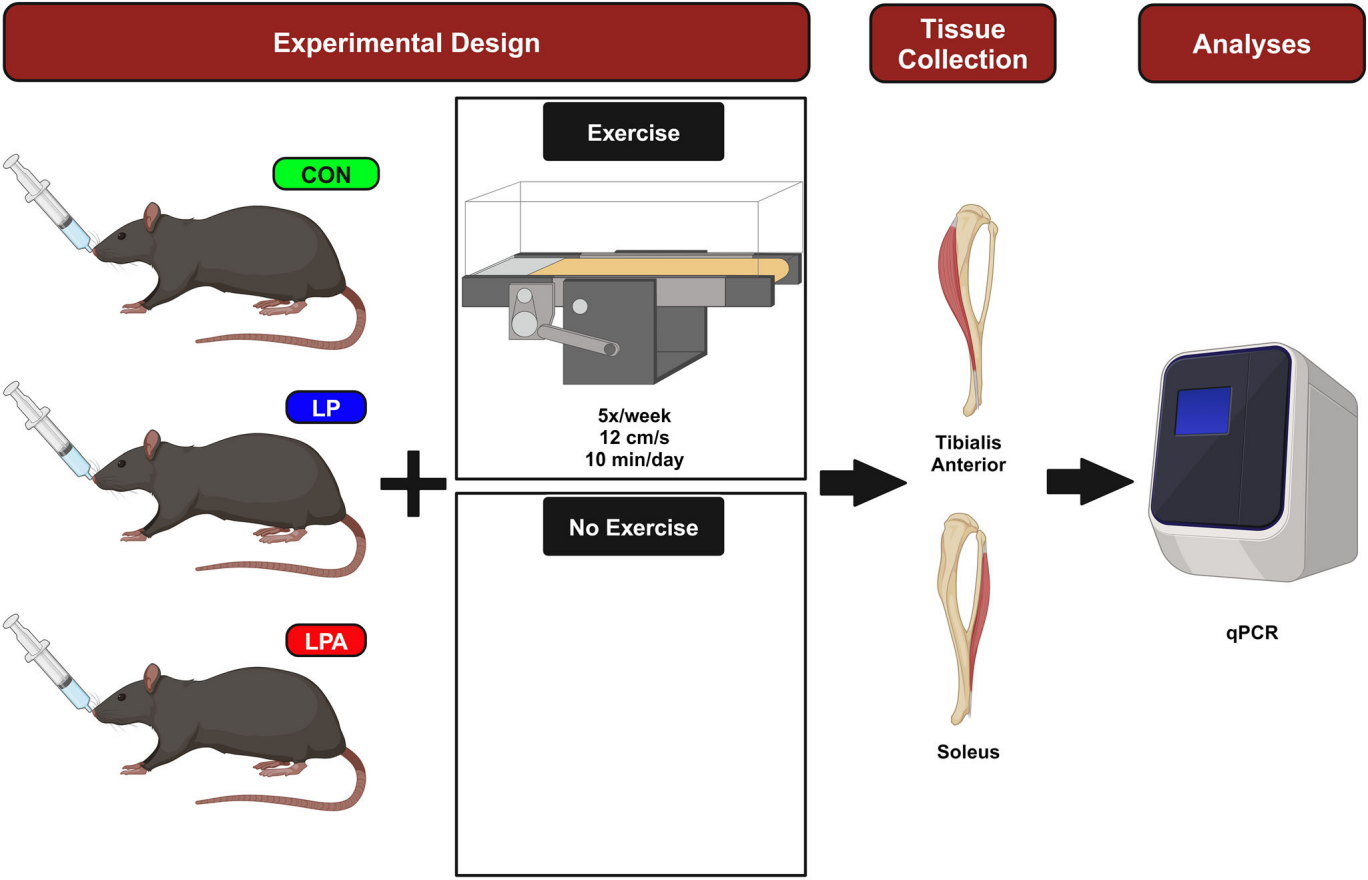
Illustration of experimental design and tissue analyses. CON, control; LP, probiotic; LPA, Ang(1–7)‐expressing probiotic; qPCR, quantitative PCR.

Exercise training sessions were conducted at 12 cm/s for 10 min/day at 0% incline, equivalent to moderate intensity exercise, for 5×/week at 09.00 h (ZT3). Of note, it is acknowledged that the exercise and probiotic interventions were conducted during the rodents’ typical rest phase. This was due to practical constraints; however, the interventions were conducted within the same time frame each day to minimise unintended outcomes and ensure consistency. All Sed groups did not perform any exercise training, but were handled and exposed to the treadmill for 10 min/day, 5×/week to reduce variance in stress between exercising groups. At the end of the intervention and following post‐intervention physical testing, rodents were euthanised via decapitation, according to NIH guidelines, and tissue was harvested immediately at 20.00 h (ZT2). Tibialis anterior and soleus muscles were collected, snap‐frozen in liquid nitrogen, and stored at −80°C until analyses. An overview of the experimental protocol is displayed in Figure 1.

### 2.3 Physical measurements

#### 2.3.1 Time to exhaustion

Procedures for time to exhaustion have been previously described. Briefly, treadmill re‐acclimatization occurred in the sedentary groups at 12 cm/s, 0% incline for 10 min at the conclusion of the intervention. On the following day, all rodents were tested for time to exhaustion beginning at 12 cm/s, 0% incline for 2 min, and increasing speed by 2 cm/s until max speed, 16 cm/s, at 09.00 h (ZT3). Time to exhaustion was recorded when the rat stopped moving and did not respond to prodding.

#### 2.3.2 Grip strength

An automated grip strength meter (Columbus Instruments, Columbus, OH, USA) was used to test forelimb grip strength. Rats were suspended by their tails over the instrument for 3 s and gently lowered towards the grip ring, where rats were able to grasp the ring with their forepaws. The animal's body was then quickly lowered to a horizontal position and tugged away from the ring until the grasp was broken. The mean force was determined by a computerised electronic pull strain gauge fitted to the grip ring. The force reported is the average of three trials relative to body weight.

### 2.4 Gene expression

Total RNA from all tissue samples was extracted using Trizol (Thermo Fisher Scientific, Waltham, MA, USA) and measured on a Nanodrop spectrophotometer (Thermo Fisher Scientific). Complementary DNA was synthesised using the SuperScript VILO cDNA synthesis kit (Thermo Fisher Scientific) from 1 µg of the resulting total RNA. The QuantStudio RealTime 5 PCR System (Thermo Fisher Scientific) was used for real‐time quantitative PCR (qPCR) detection at 40 cycles in duplicate. TaqMan primers were used for analyses and are listed in Table 2. *Actb* was used as an endogenous control for comparative data of target genes, and data were quantifiably analysed via the comparative *C*_t_ (ΔΔ*C*_t_) method. Experimental group data were normalised to CON Sed values.

**TABLE 2:** Quantitative PCR (qPCR) primer targets.

**Target gene**	**Thermo Fisher Scientific assay ID**
Actin beta (*Actb*)	Rn00667869_m1
Brain and muscle ARNT like 1 (*BMAL1*)	Rn00577590_m1
Circadian locomotor output cycles kaput (*CLOCK*)	Rn01413228_m1
Cryptochrome circadian regulator 1 (*CRY1*)	Rn01503063_m1
Cryptochrome circadian regulator 2 (*CRY2*)	Rn01485701_m1
D‐box binding PAR BZIP transcription factor (*DBP*)	Rn01498425_m1
Nuclear factor, interleukin 3 regulated (*NFIL3*)	Rn01434874_s1
Neuronal PAS domain protein 2 (*NPAS2*)	Rn01438223_m1
Nuclear receptor subfamily 1 group D member 1 (*NR1D1*)	Rn01460662_m1
Nuclear receptor subfamily 1 group D member 2 (*NR1D2*)	Rn00596011_m1
Period circadian regulator (*PER1*)	Rn01325256_m1
Period circadian regulator (*PER2*)	Rn01427704_m1

### 2.5 Statistical analyses

Data are expressed as means ± standard deviation (SD). A log‐rank Kaplan–Meier curve was performed to assess survival between groups. A repeated measures ANOVA was performed to analyse pre‐ and post‐weight within groups. A one‐way ANOVA was performed to analyse changes in weight between groups. A two‐way ANOVA was performed for individual gene expression comparisons between intervention groups, including interaction effects for oral treatment and exercise condition in both tissues in each sex. Additionally, we explored potential differences of LP and LPA relative to CON, within each sex and exercise condition, using pre‐planned contrasts. Non‐normalised data were analysed by Kruskal–Wallis nonparametric testing. Pearson correlation coefficients were utilised to examine the relationship between differentially expressed transcripts and statistically significant performance measures. The correlations were classified as weak (|*r*| < 0.3), moderate (|*r*| ≥ 0.3 and |*r*| < 0.8) or large (|*r*| ≥ 0.8). SPSS v29.0 (IBM Corp., Armonk, NY, USA) was used to perform statistical analyses. Statistical significance was determined at *P* < 0.05. For contrast comparisons, we did not adjust the *P*‐value for multiple comparisons; however, in order to facilitate interpretation, significance is reported at three different values – *P* < 0.05, *P* < 0.01 and *P* < 0.001.

## 3 RESULTS

### 3.1 Physical measurements and survival

**FIGURE 2:**
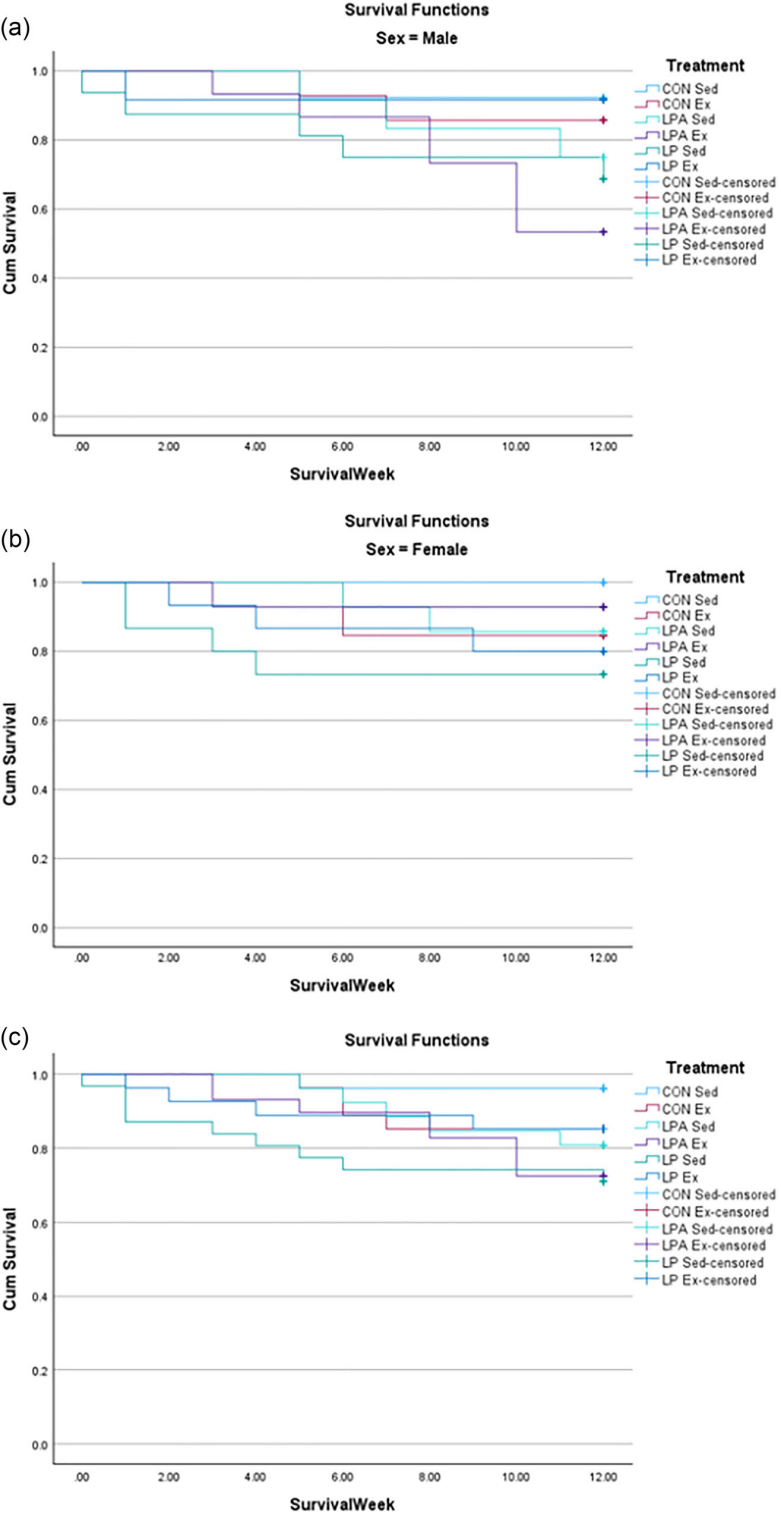
Kaplan–Meier survival curve for male rats (a), female rats (b), and overall (c).

**FIGURE 3:**
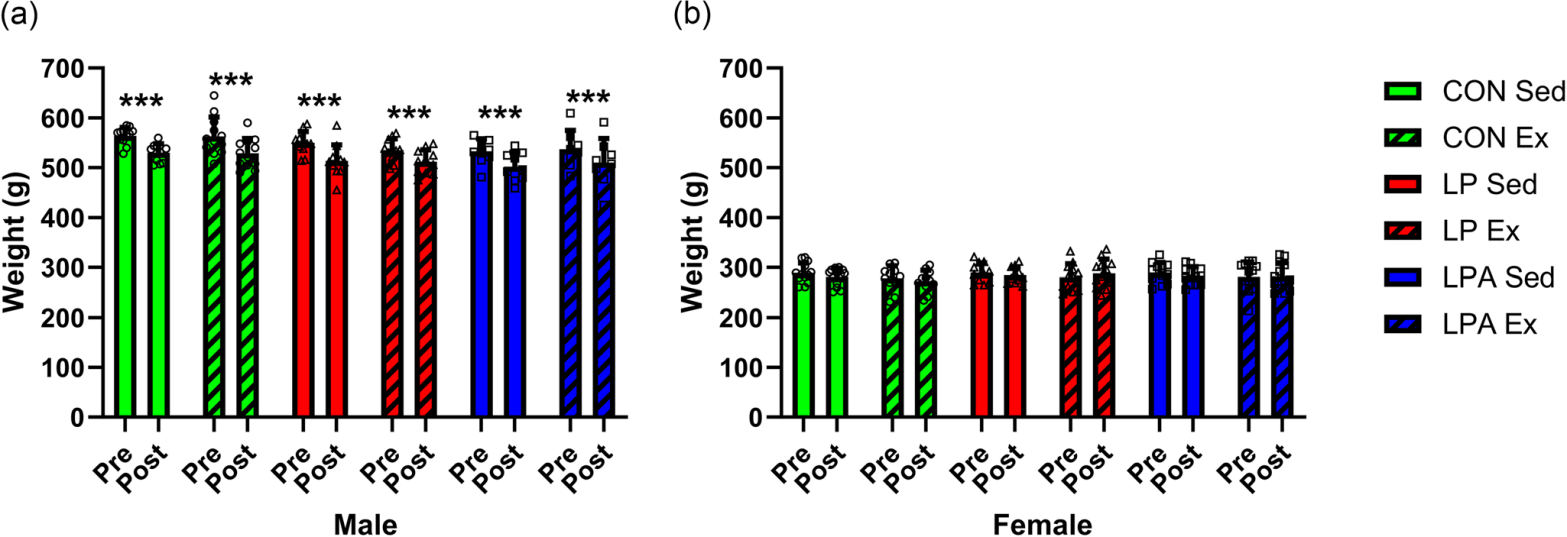
Changes in body weight (g) for male (a) and female (b) rats per group. Data are presented as means ± SD. ^***^P < 0.001. CON, control (*n* = 24, male; *n* = 24, female); Ex, exercise (*n* = 31, male; *n* = 36, female); LP, probiotic (*n* = 22, male; *n* = 23, female); LPA, Ang(1–7)‐expressing probiotic (*n* = 17, male; *n* = 25, female); Sed, sedentary (*n* = 32, male; *n* = 36, female).

**FIGURE 4:**
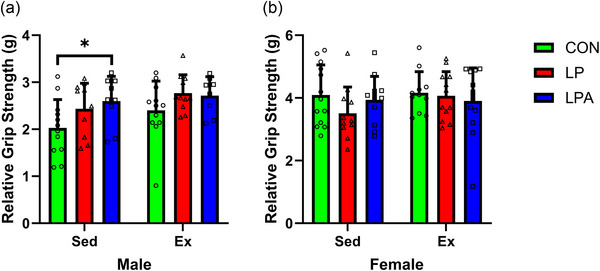
Relative grip strength (g) for male (a) and female (b) rats per group. Data are presented as means ± SD. ^*^P < 0.05. CON, control (*n* = 24, male; *n* = 24, female); Ex, exercise (*n* = 31, male; *n* = 36, female); LP, probiotic (*n* = 22, male; *n* = 23, female); LPA, Ang(1–7)‐expressing probiotic (*n* = 17, male; *n* = 25, female); Sed, sedentary (*n* = 32, male; *n* = 36, female).

For males, the survival percentages for each group were as follows: 92.3% for CON Sed, 85.7% for CON Ex, 62.5% for LP Sed, 91.7% for LP Ex, 75% for LPA Sed and 53.3% for LPA Ex. No significant difference was found in males between groups pertaining to survival (*P* = 0.090; Figure 2a). For females, the survival percentages for each group were as follows: 84.6% for CON Sed, 84.6% for CON Ex, 66.7% for LP Sed, 80% for LP Ex, 71.4% for LPA Sed and 78.6% for LPA Ex. No significant difference was found in females between groups pertaining to survival (*P* = 0.765; Figure 2b). Alternatively, no significant difference was found for survival between males (76.8%) and females (85.7%; *P* = 0.153). The survival percentages for each group for males and females combined were as follows: 96.2% for CON Sed, 85.2% for CON Ex, 71% for LP Sed, 85.2% for LP Ex, 80.8% for LPA Sed and 72.4% for LPA Ex. Overall, no significant difference between groups was found when combining males and females pertaining to survival (*P* = 0.149; Figure 2c). Pre and post body weight are displayed in Figure 3. In males only, the main effect of oral treatment was found for pre vs. post body weight (*P* = 0.010). Subsequently, body weight decreased in all groups across the study (*P* < 0.001). However, no weight change between groups was significant. Alternatively, no main effect was found for pre vs. post body weight, and no changes in body weight within groups were significant in females. Time to exhaustion results for both male and female rats have been previously reported (Hernandez et al., 2022) and are available for review in Supporting information, Table S1. Briefly, male rats increased the time to exhaustion as a result of exercise training, but no difference between groups was found. The female rats demonstrated a longer time to exhaustion in the LPA + Ex group compared to the CON + Ex group as a result of both the probiotic intervention and exercise training. However, no interaction effect was found for the female rats. Figure 4 displays the relative grip strength for male and female rats. A main effect of oral treatment was found for relative grip strength only for male rats (*P* = 0.034). Furthermore, the CON Sed group's relative grip strength was lower compared to LPA Sed (*P* = 0.018). No main effect was found for females in relation to relative grip strength (oral treatment, *P* = 0.398; exercise, *P* = 0.333). Therefore, no correlative statistics were performed for females in relation to relative grip strength.

### 3.2 Differential expression of circadian rhythm targets in males

#### 3.2.1 Tibialis anterior

**FIGURE 5:**
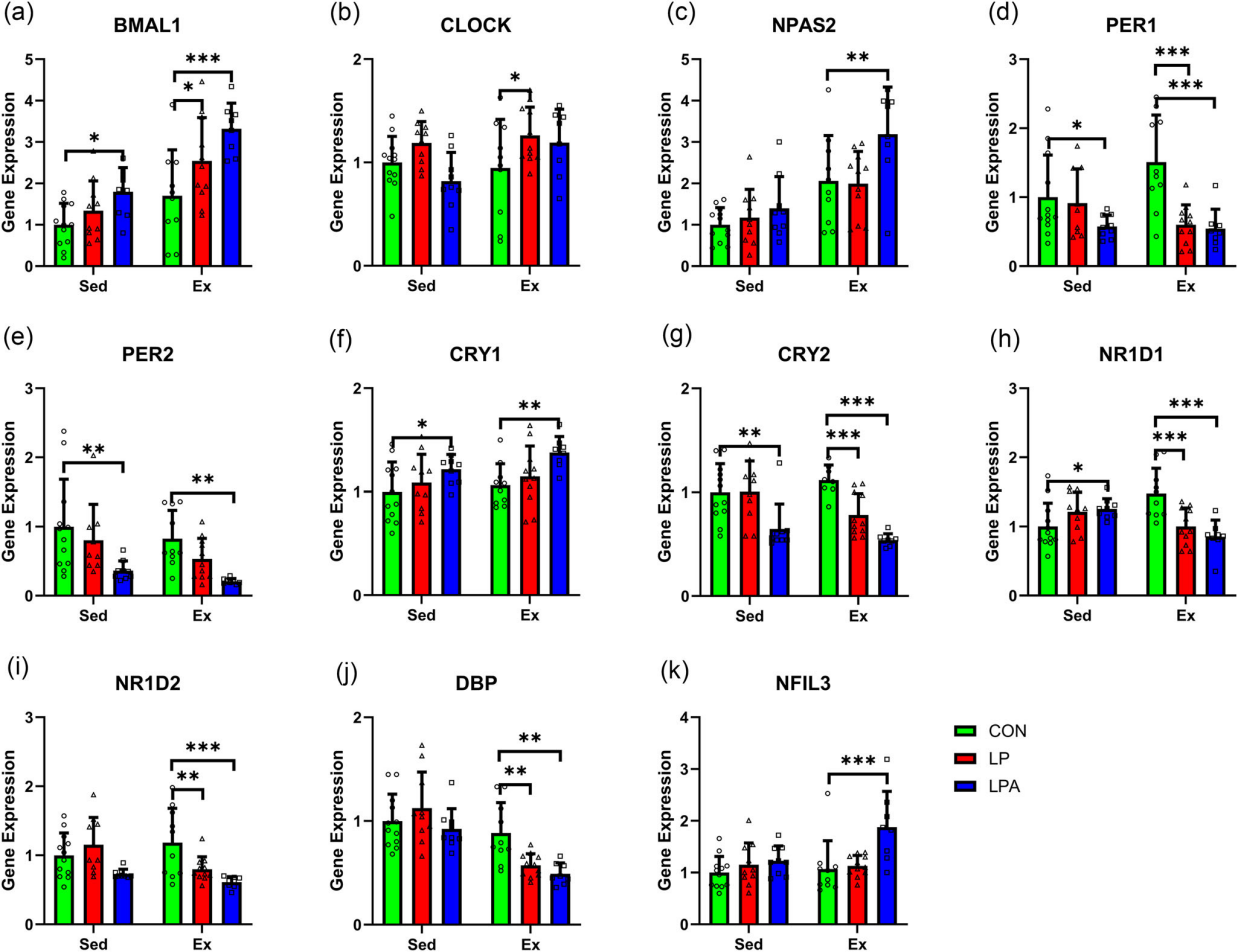
Skeletal muscle tibialis anterior circadian rhythms in gene expression of male rats. Difference in gene expression between all groups for *BMAL1* (a), *CLOCK* (b), *NPAS2* (c), *PER1* (d), *PER2* (e), *CRY1* (f), *CRY2* (g), *NRID1* (h), *NR1D2* (i), *DBP* (j), and *NFIL3* (k). Data are presented as means ± SEM. ^*^ *P* < 0.05, ^**^ *P* < 0.01, ^***^ *P* < 0.001. CON, control (*n* = 22); Ex, exercise (*n* = 29); LP, probiotic (*n* = 21); LPA, Ang(1–7)‐expressing probiotic (*n* = 17); Sed, sedentary (*n* = 31).

An interaction effect between exercise and oral treatment was found for *NR1D1* expression only (*P* < 0.001; Figure 5h). A main effect of oral treatment significantly affected the expression of genes *BMAL1* (*P* < 0.001; Figure 5a), *CLOCK* (*P* = 0.023; Figure 5b), *PER1* (*P* = 0.002; Figure 5d), *PER2* (*P* < 0.001; Figure 5e), *CRY1* (*P* = 0.005; Figure 5f), *CRY2* (*P* < 0.001; Figure 5g), *NR1D2* (*P* < 0.001; Figure 5i) and *NFIL3* (*P* = 0.003; Figure 5k). Lastly, a main effect of exercise significantly affected the expression of *BMAL1* (*P* < 0.001), *NPAS2* (*P* < 0.001; Figure 5c) and *DBP* (*P* < 0.001; Figure 5j).

Irrespective of exercise group, LPA increased mRNA expression of *BMAL1* (*P *< 0.001), *NPAS2* (*P* = 0.006), *CRY1* (*P* < 0.001) and *NFIL3* (*P* < 0.001) and decreased expression of *PER1* (*P* < 0.001), PER2 (*P* < 0.001), *CRY2* (*P* < 0.001), *NR1D2* (*P* < 0.001) and *DBP* (*P* = 0.004) compared to CON. In comparison, LP increased mRNA expression of *BMAL1* (*P* = 0.020) and *CLOCK* (*P* = 0.010) only and decreased expression of *PER1* (*P* = 0.001) and *CRY2* (*P* = 0.027) only compared to CON (Figure 5).

In the sedentary group, LPA increased mRNA expression of *BMAL1* (*P* = 0.029), *CRY1* (*P *= 0.047) and *NR1D1* (*P* = 0.049) and decreased expression of *PER1* (*P* = 0.047), *PER2* (*P* = 0.002) and *CRY2* (*P* = 0.001) compared to CON. Meanwhile, LP did not influence mRNA expression of any gene in the sedentary group compared to CON.

In the exercise group, LPA increased mRNA expression of *BMAL1* (*P* < 0.001), *NPAS2* (*P* = 0.006), *CRY1* (*P* = 0.008) and *NFIL3* (*P* < 0.001), and decreased expression of *PER1* (*P* < 0.001), *PER2* (*P* = 0.004), *CRY2* (*P* < 0.001), *NR1D1* (*P* < 0.001), *NR1D2* (*P* < 0.001) and *DBP* (*P* = 0.001) compared to CON. Meanwhile, LP increased mRNA expression of *BMAL1* (*P* = 0.020) and *CLOCK* (*P* = 0.024) and decreased expression of *PER1* (*P* < 0.001), *CRY2* (*P* = 0.003), *NR1D1* (*P* < 0.001), *NR1D2* (*P* = 0.006) and *DBP* (*P* = 0.004) compared to CON.

#### 3.2.2 Soleus

**FIGURE 6:**
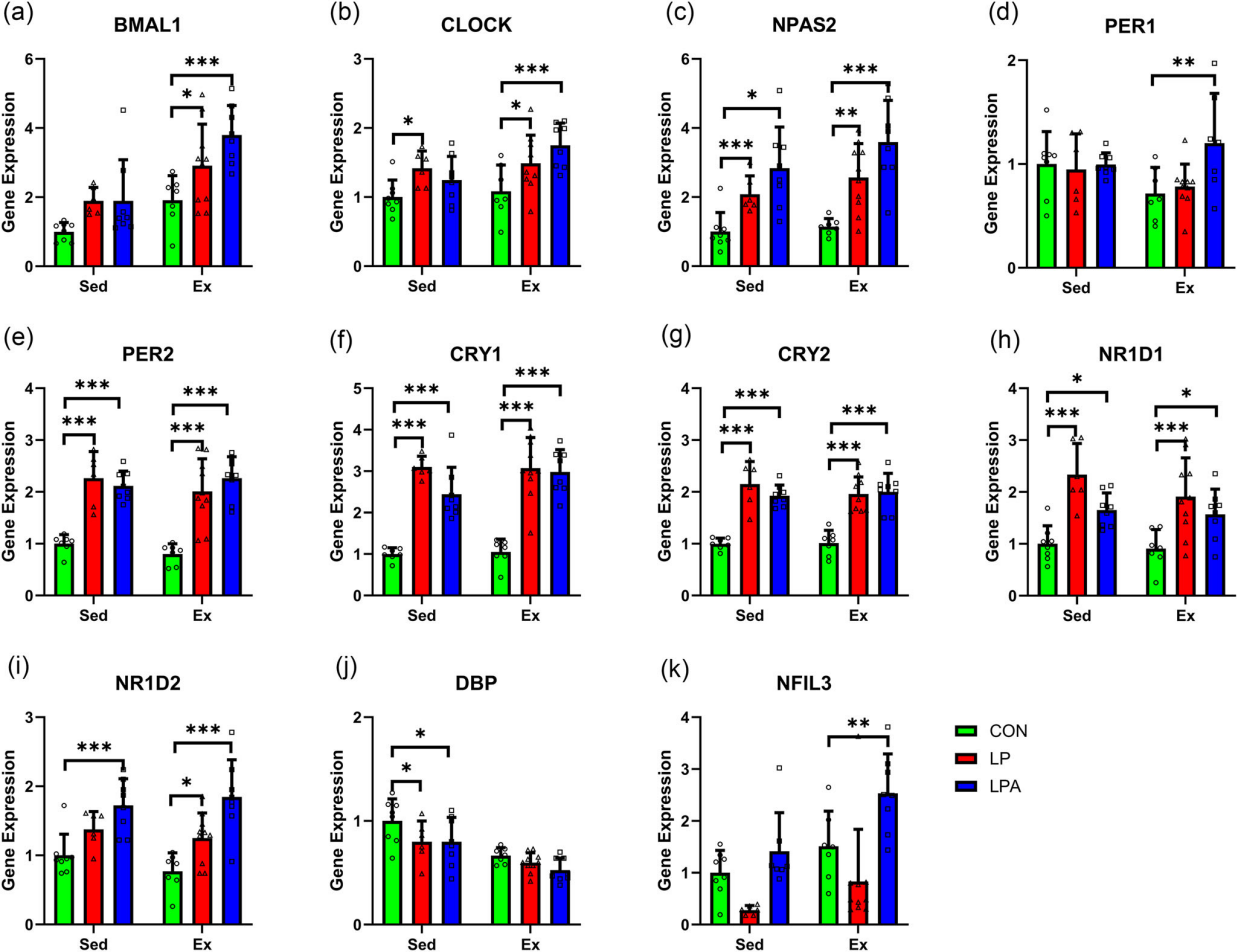
Skeletal muscle soleus circadian rhythms gene expression of male rats. Difference in gene expression between all groups for *BMAL1* (a), *CLOCK* (b), *NPAS2* (c), *PER1* (d), *PER2* (e), *CRY1* (f), *CRY2* (g), *NRID1* (h), *NR1D2* (i), *DBP* (j), and *NFIL3* (k). Data are presented as means ± SEM. ^*^ *P* < 0.05, ^**^ *P* < 0.01, ^***^ *P* < 0.001. CON, control (*n* = 15); Ex, exercise (*n* = 25); LP, probiotic (*n* = 16); LPA, Ang(1–7)‐expressing probiotic (*n* = 16); Sed, sedentary (*n* = 22).

No interaction effect between exercise and oral treatment was found for any gene analysed, nor was any main effect found for *PER1*. A main effect of oral treatment significantly affected the expression of genes *BMAL1* (*P* = 0.002; Figure 6a), *CLOCK* (*P* = 0.001; Figure 6b), *NPAS2* (*P* < 0.001; Figure 6c), *PER2* (*P* < 0.001; Figure 6e), *CRY1* (*P* < 0.001; Figure 6f), *CRY2* (*P* < 0.001; Figure 6g), *NR1D1* (*P* < 0.001; Figure 6h), *NR1D2* (*P* < 0.001; Figure 6i), *DBP* (*P* = 0.046; Figure 6j) and *NFIL3* (*P* < 0.001; Figure 6k). A main effect of exercise significantly affected the expression of genes *BMAL1* (*P* < 0.001), *CLOCK* (*P* = 0.034), *DBP* (*P* < 0.001) and *NFIL3* (*P* = 0.047).

Irrespective of exercise group, LPA increased mRNA expression of *BMAL1* (*P* < 0.001), *CLOCK* (*P* = 0.001), *NPAS2* (*P* < 0.001), *PER1* (*P* = 0.033), *PER2* (*P* < 0.001), *CRY1* (*P* < 0.001), *CRY2* (*P* < 0.001), *NR1D1* (*P* = 0.001), *NR1D2* (*P* < 0.001) and *NFIL3* (*P* = 0.009) and decreased mRNA expression of *DBP* (*P* = 0.006) only compared to CON. In comparison, LP increased mRNA expression of *BMAL1* (*P* = 0.006), *CLOCK* (*P* = 0.002), *NPAS2* (*P* < 0.001), *PER2* (*P* < 0.001), *CRY1* (*P* < 0.001), *CRY2* (*P* < 0.001), *NR1D1* (*P* < 0.001), *NR1D2* (*P* = 0.003) and *NFIL3* (*P* = 0.011) and decreased mRNA expression of *DBP* (*P* = 0.032) only (Figure 6).

In the sedentary group, LPA increased mRNA expression of *NPAS2* (*P* < 0.001), *PER2* (*P* < 0.001), *CRY1* (*P* < 0.001), *CRY2* (*P* < 0.001), *NR1D1* (*P* = 0.016) and *NR1D2* (*P* < 0.001) and decreased mRNA expression of *DBP* only (*P* = 0.019) compared to CON. Meanwhile, LP increased mRNA expression of *CLOCK* (*P* = 0.028), *NPAS2* (*P* = 0.030), *PER2* (*P* < 0.001), *CRY1* (*P* < 0.001), *CRY2* (*P* < 0.001) and *NR1D1* (*P* < 0.001) and decreased mRNA expression of *DBP* only (*P* = 0.030) compared to CON.

In the exercise group, LPA increased mRNA expression of *BMAL1* (*P* < 0.001), *CLOCK* (*P* < 0.001), *NPAS2* (*P* < 0.001), *PER1* (*P* = 0.004), *PER2* (*P* < 0.001), *CRY1* (*P* < 0.001), *CRY2* (*P* < 0.001), *NR1D1* (*P* = 0.017), *NR1D2* (*P* < 0.001) and *NFIL3* (*P* = 0.009) compared to CON. Meanwhile, LP increased mRNA expression of *BMAL1* (*P* = 0.028), *CLOCK* (*P* = 0.019), *NPAS2* (*P* = 0.002), *PER2* (*P* < 0.001), *CRY1* (*P* < 0.001), *CRY2* (*P* < 0.001), *NR1D1* (*P* < 0.001) and *NR1D2* (*P* = 0.012) compared to CON.

### 3.3 Relationship of circadian rhythms and physical function in males

#### 3.3.1 Tibialis anterior

**FIGURE 7:**
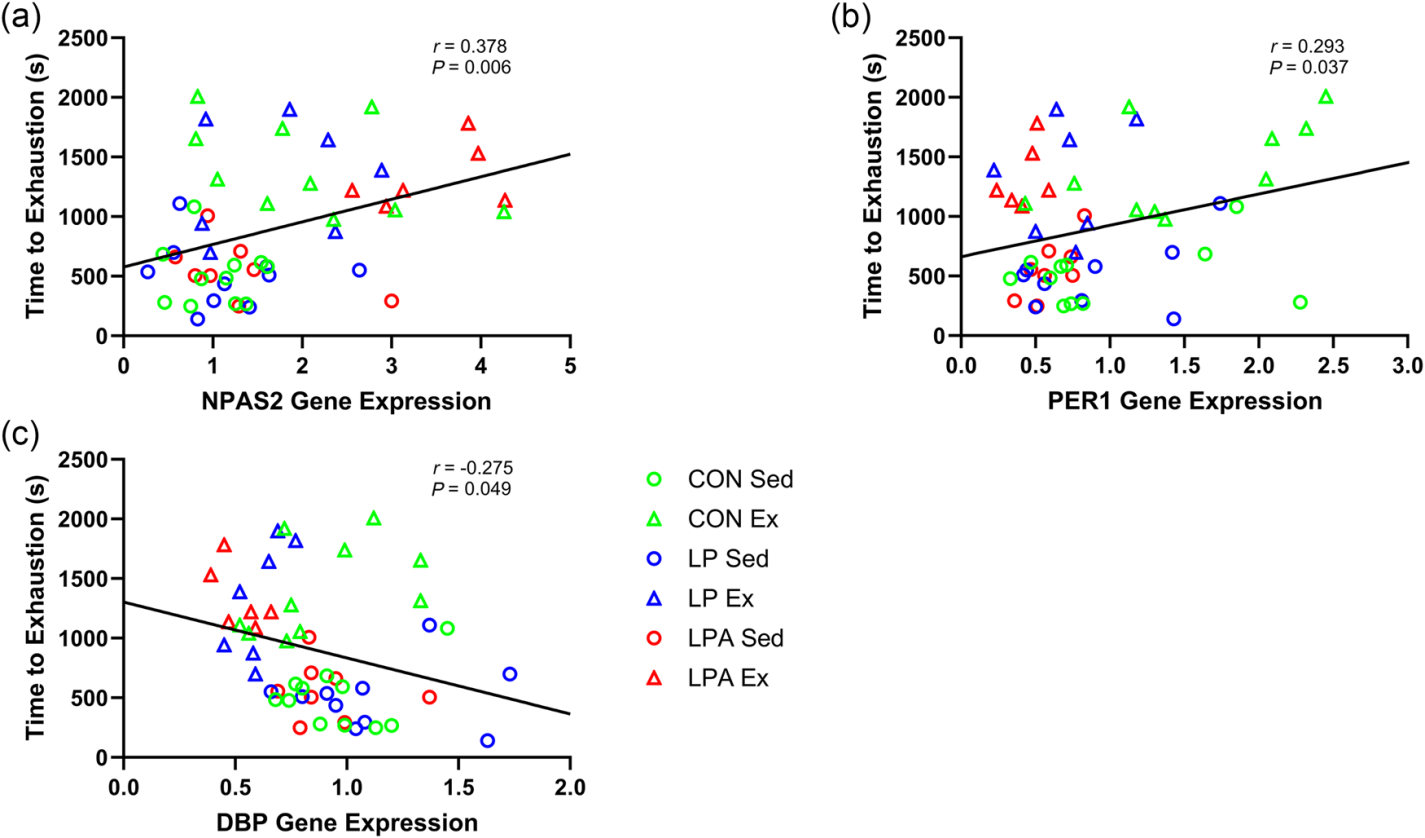
Skeletal muscle tibialis anterior Pearson correlation examining the relationship of time to exhaustion with individual gene expression of male rats. Only significant correlations are displayed in the figure. Relationship of time to exhaustion with *NPAS2* (a), *PER1* (b), and *DBP* (c). CON Sed, control + sedentary group (*n* = 11); CON Ex, control + exercise group (*n* = 10); LP Sed, probiotic + sedentary group (*n* = 10); LP Ex, probiotic + exercise group (*n* = 7); LPA Sed, Ang(1–7)‐expressing probiotic + sedentary group (*n* = 8); LPA Ex, Ang(1–7)‐expressing probiotic + exercise group (*n* = 6).

**FIGURE 8:**
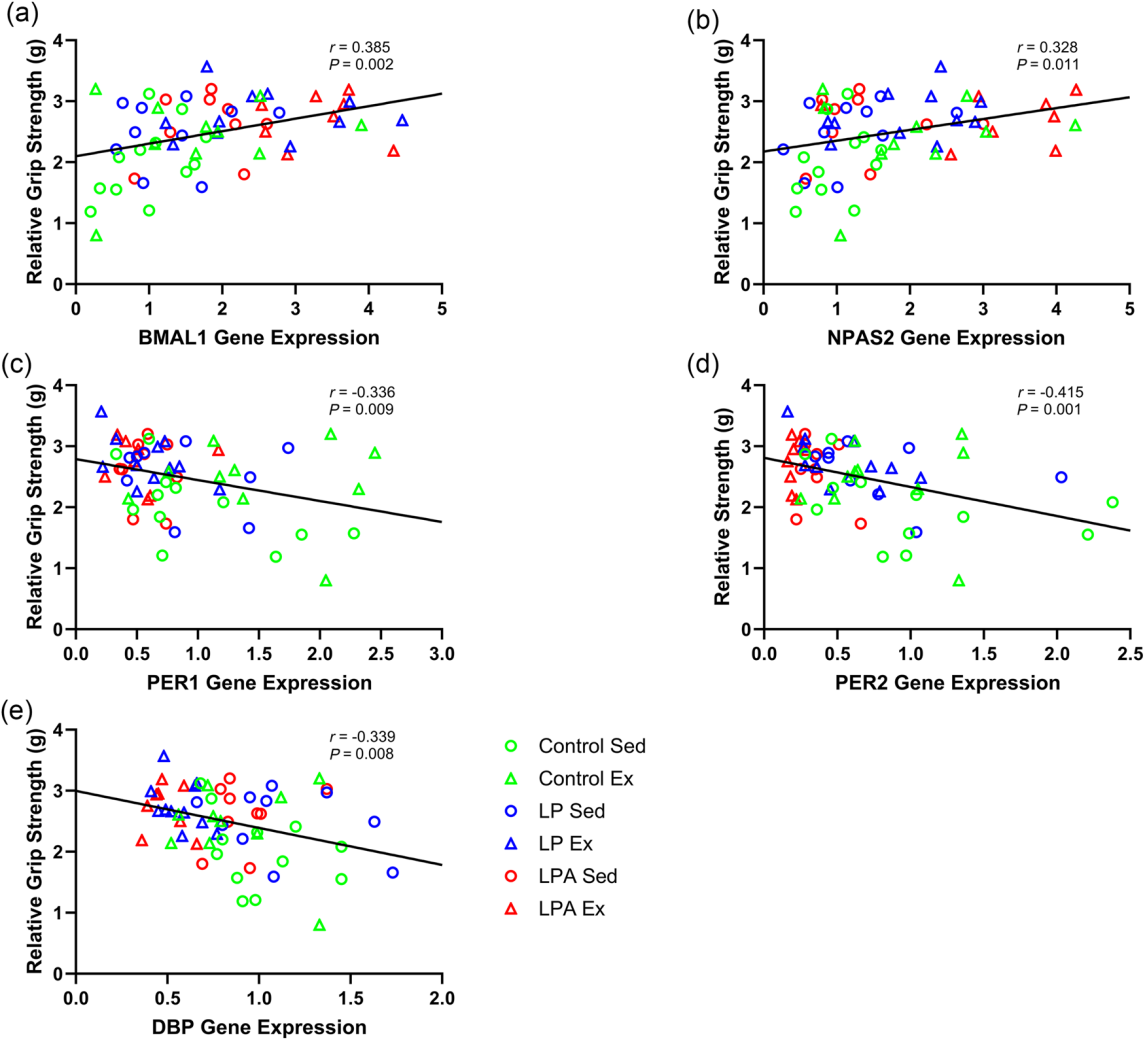
Skeletal muscle tibialis anterior correlation examining the relationship of relative grip strength with individual gene expression of male rats. Only significant correlations are displayed in the figure. Relationship of relative grip strength with *BMAL1* (a), *NPAS2* (b), *PER1* (c), *PER2* (d), and *DBP* (e). CON Sed, control + sedentary group (*n* = 12); CON Ex, control + exercise group (*n* = 10); LP Sed, probiotic + sedentary group (*n* = 10); LP Ex, probiotic + exercise group (*n* = 11); LPA Sed, Ang(1–7)‐expressing probiotic + sedentary group (*n* = 9); LPA Ex, Ang(1–7)‐expressing probiotic + exercise group (*n* = 8).

There was a weak positive correlation between *NPAS2* expression and time to exhaustion (Figure 7a). *PER1* expression also had a weak positive correlation with time to exhaustion (Figure 7b). Additionally, *DBP* expression had a weak negative correlation with time to exhaustion (Figure 7c). There was a modest positive correlation for *BMAL1* expression (Figure 8a), a modest positive correlation for *NPAS2* expression (Figure 8b), a modest negative correlation for *PER1* expression (Figure 8c), a modest negative correlation for *PER2* expression (Figure 8d), and a modest negative correlation for *DBP* expression (Figure 8e) with relative grip strength.

#### 3.3.2 Soleus

**FIGURE 9:**
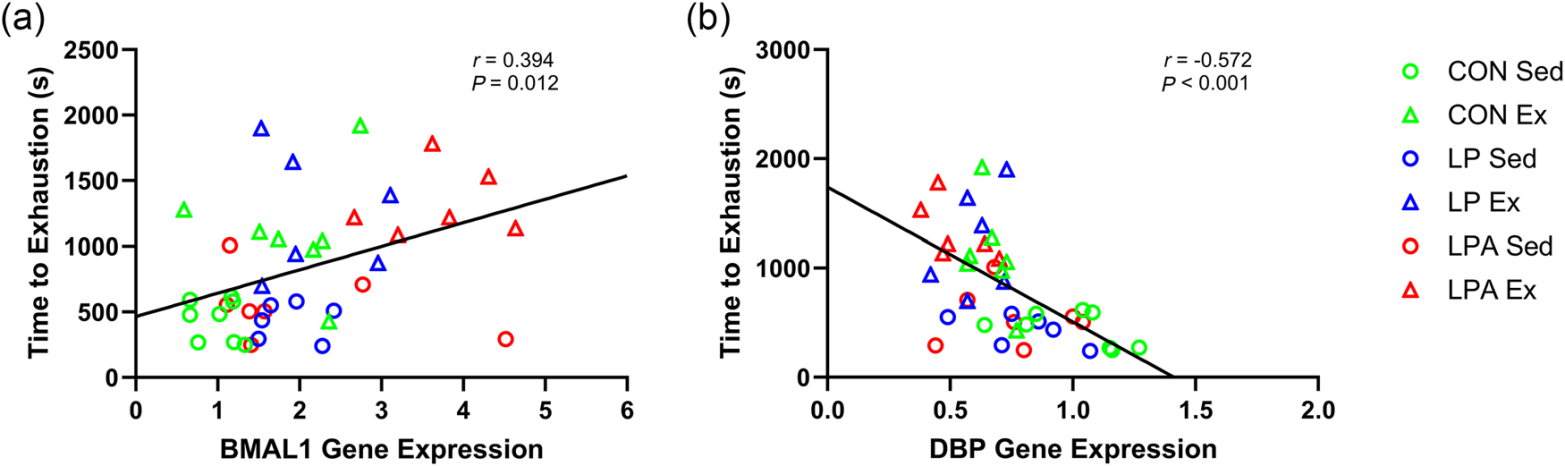
Skeletal muscle soleus correlation examining the relationship of time to exhaustion with individual gene expression of male rats. Only significant correlations are displayed in the figure. Relationship of time to exhaustion with *BMAL1* (a) and *DBP* (b). CON Sed, control + sedentary group (*n* = 8); CON Ex, control + exercise group (*n* = 7); LP Sed, probiotic + sedentary group (*n* = 6); LP Ex, probiotic + exercise group (*n* = 6); LPA Sed, Ang(1–7)‐expressing probiotic + sedentary group (*n* = 7); LPA Ex, Ang(1–7)‐expressing probiotic + exercise group (*n* = 6).

**FIGURE 10:**
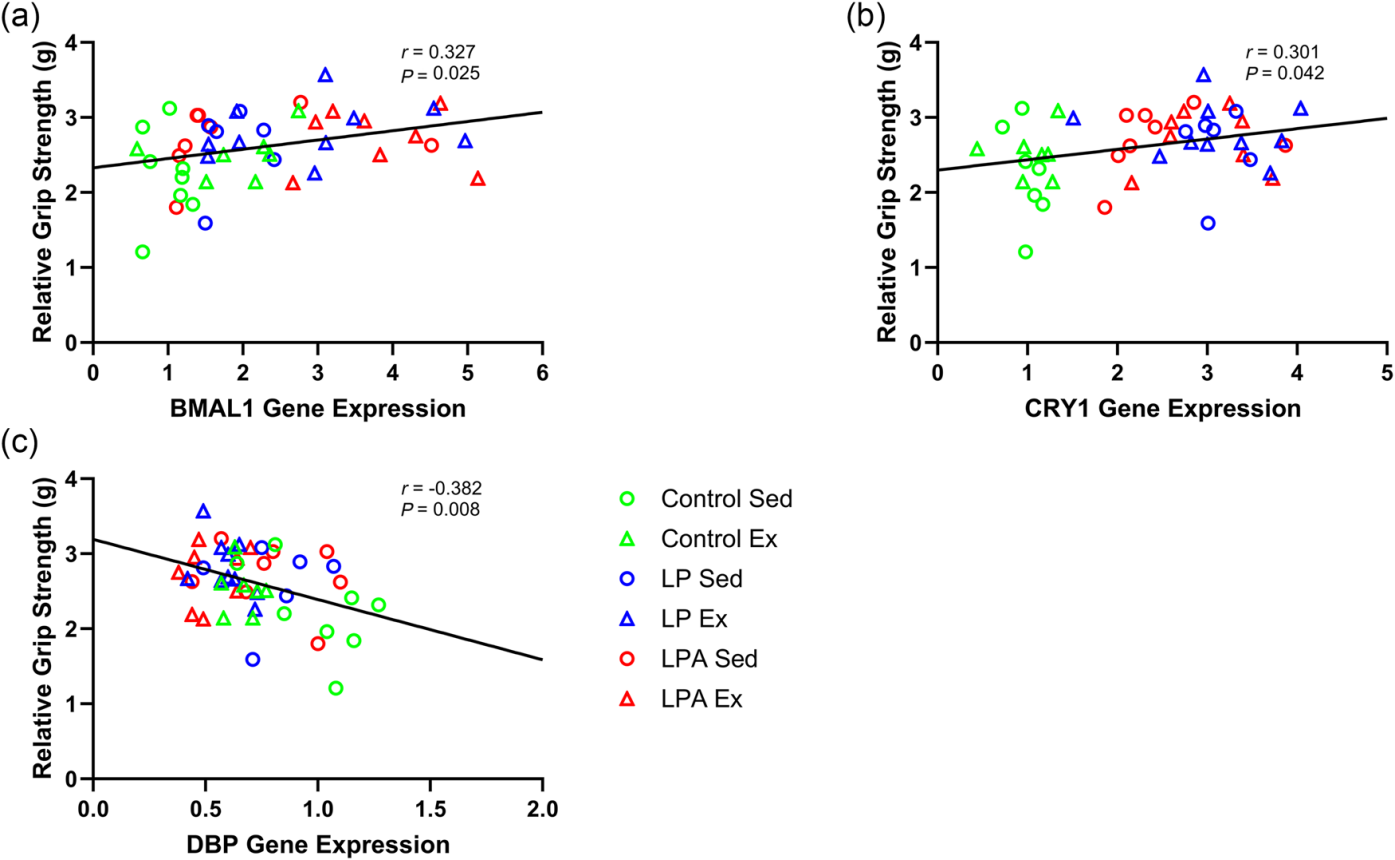
Skeletal muscle soleus correlation examining the relationship of relative grip strength with individual gene expression of male rats. Only significant correlations are displayed in the figure. Relationship of relative grip strength with *BMAL1* (a), *CRY1* (b), and *DBP* (c). CON Sed, control + sedentary group (*n* = 8); CON Ex, control + exercise group (*n* = 7); LP Sed, probiotic + sedentary group (*n* = 6); LP Ex, probiotic + exercise group (*n* = 6); LPA Sed, Ang(1–7)‐expressing probiotic + sedentary group (*n* = 9); LPA Ex, Ang(1–7)‐expressing probiotic + exercise group (*n* = 8).

There was a weak positive correlation between *BMAL1* expression and time to exhaustion (Figure 9a). Additionally, *DBP* expression had a moderate negative correlation with time to exhaustion (Figure 9b). There was a modest positive correlation for *BMAL1* expression (Figure 10a), a modest positive correlation for *CRY1* expression (Figure 10b), and a modest negative correlation for *DBP* expression (Figure 10c) with relative grip strength.

### 3.4 Differential expression of circadian rhythms targets in females

#### 3.4.1 Tibialis anterior

**FIGURE 11:**
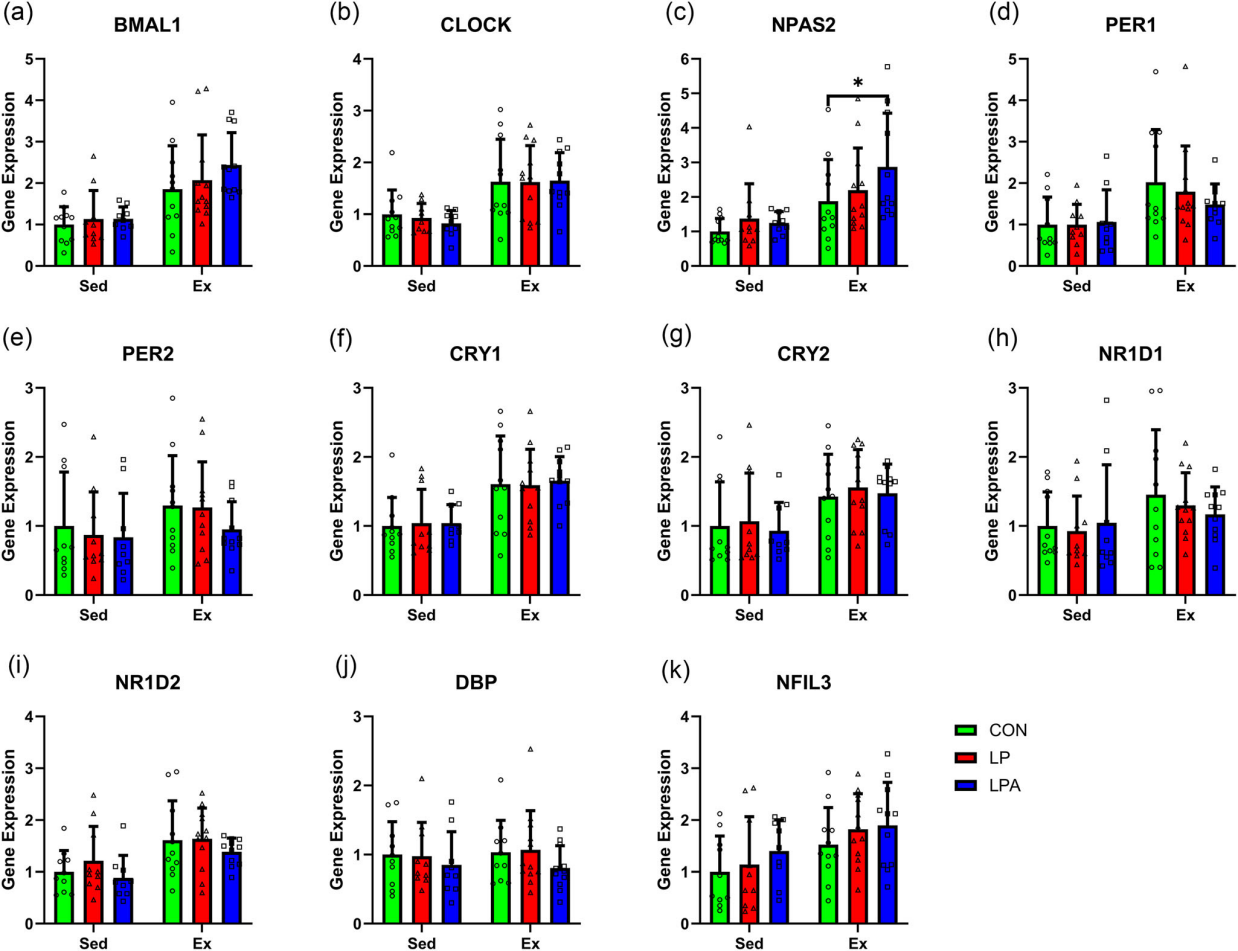
Skeletal muscle tibialis anterior circadian rhythms in gene expression of female rats. Difference in gene expression between all groups for *BMAL1* (a), *CLOCK* (b), *NPAS2* (c), *PER1* (d), *PER2* (e), *CRY1* (f), *CRY2* (g), *NRID1* (h), *NR1D2* (i), *DBP* (j), and *NFIL3* (k). Data are presented as means ± SEM. *P < 0.05. CON, control (*n* = 22); Ex, exercise (*n* = 34); LP, probiotic (*n* = 22); LPA, Ang(1–7)‐expressing probiotic (*n* = 20); Sed, sedentary (*n* = 30).

No interaction effect nor main effect of oral treatment significantly affected the expression of any gene analysed, nor did either main effect significantly alter the expression of *DBP* (Figure 11j). A main effect of exercise significantly affected the expression of *BMAL1* (*P* < 0.001; Figure 11a), *CLOCK* (*P* < 0.001; Figure 11b), *NPAS2* (*P* < 0.001; Figure 11c), *PER1* (*P* < 0.001; Figure 11d), *PER2* (*P* = 0.015; Figure 11e), *CRY1* (*P* < 0.001; Figure 11f), *CRY2* (*P* = 0.001; Figure 11g), *NR1D1* (*P* = 0.021; Figure 11h), *NR1D2* (*P* < 0.001; Figure 11i) and *NFIL3* (*P* = 0.004; Figure 11k).

Irrespective of exercise group, LPA or LP did not influence mRNA expression of any gene compared to CON (Figure 11).

In the exercise group, LPA increased mRNA expression of *NPAS2* only (*P* = 0.034) compared to CON. LP did not influence mRNA expression of any gene compared to CON in the exercise group.

Neither LPA nor LP influenced mRNA expression compared to CON in the sedentary group.

#### 3.4.2 Soleus

**FIGURE 12:**
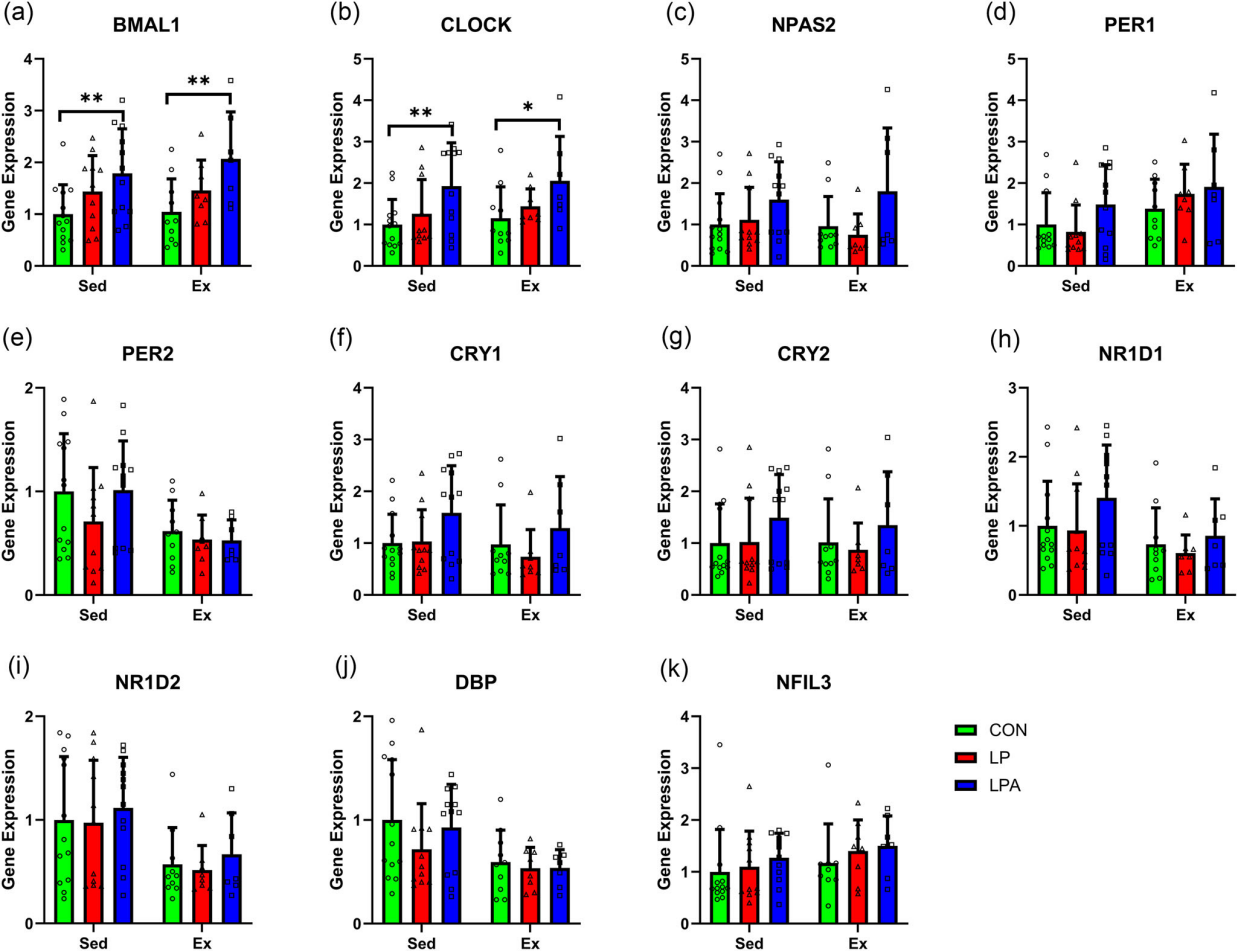
Skeletal muscle soleus circadian rhythms gene expression of female rats. Difference in gene expression between all groups for *BMAL1* (a), *CLOCK* (b), *NPAS2* (c), *PER1* (d), *PER2* (e), *CRY1* (f), *CRY2* (g), *NRID1* (h), *NR1D2* (i), *DBP* (j), and *NFIL3* (k). Data are presented as means ± SEM. ^*^ *P* < 0.05, ^**^ *P* < 0.01. CON, control (*n* = 23); Ex, exercise (*n* = 25); LP, probiotic (*n* = 18); LPA, Ang(1–7)‐expressing probiotic (*n* = 19); Sed, sedentary (*n* = 36).

No interaction effect nor main effect of oral treatment significantly affected the expression of any gene analysed, nor did either main effect significantly alter the expression of *NPAS2* (Figure 12c), *CRY1* (Figure 12f), *CRY2* (Figure 12g) and *NFIL3* (Figure 12k). A main effect of oral treatment significantly affected the expression of *BMAL1* (*P* = 0.002; Figure 12a) and *CLOCK* (*P* = 0.005; Figure 12b). Moreover, a main effect of exercise significantly affected the expression of *PER1* (*P* = 0.017; Figure 12d), *PER2* (*P* = 0.011; Figure 12e), *NR1D1* (*P* = 0.021; Figure 12h), *NR1D2* (*P* = 0.003; Figure 12i) and *DBP* (*P* = 0.015; Figure 12j).

Irrespective of exercise group, LPA increased mRNA expression of *BMAL1* (*P* < 0.001), *CLOCK* (*P* = 0.001) and *NPAS2* (*P* = 0.012) only compared to CON. LP did not influence mRNA expression of any gene compared to CON (Figure 12).

In the sedentary group, LPA increased mRNA expression of *BMAL1* (*P* = 0.008) and *CLOCK* (*P* = 0.006) compared to CON. Meanwhile, LP did not influence mRNA expression of any gene in the sedentary group compared to CON.

In the exercise group, LPA increased mRNA expression of *BMAL1* (*P* = 0.005) and *CLOCK* (*P* = 0.028) compared to CON. LP, however, did not influence mRNA expression of any gene in the exercise group compared to CON.

### 3.5 Relationship of circadian rhythms and physical function in females

#### 3.5.1 Tibialis anterior

**FIGURE 13:**
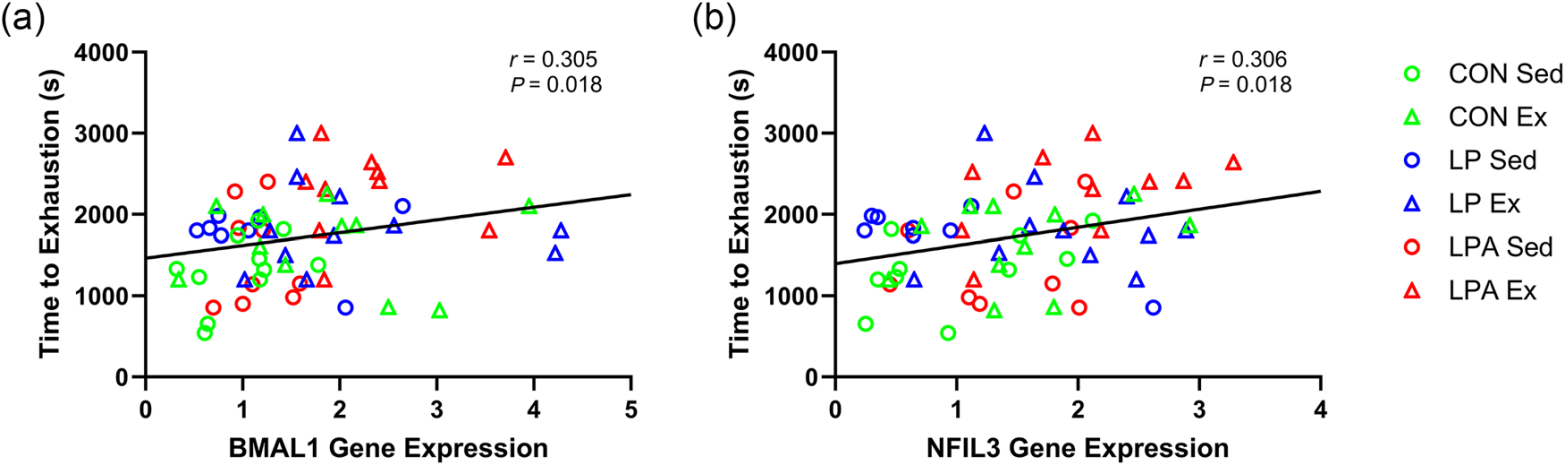
Skeletal muscle tibialis anterior correlation examining the relationship of time to exhaustion with individual gene expression of female rats. Relationship of time to exhaustion with *BMAL1* (a) and *NFIL3* (b). CON Sed, control + sedentary group (*n* = 11); CON Ex, control + exercise group (*n* = 11); LP Sed, probiotic + sedentary group (*n* = 8); LP Ex, probiotic + exercise group (*n* = 11); LPA Sed, Ang(1–7)‐expressing probiotic + sedentary group (*n* = 9); LPA Ex, Ang(1–7)‐expressing probiotic + exercise group (*n* = 10).

There was a weak positive correlation for both *BMAL1* expression (Figure 13a) and *NFIL3* expression (Figure 13b) with time to exhaustion.

#### 3.5.2 Soleus

**FIGURE 14:**
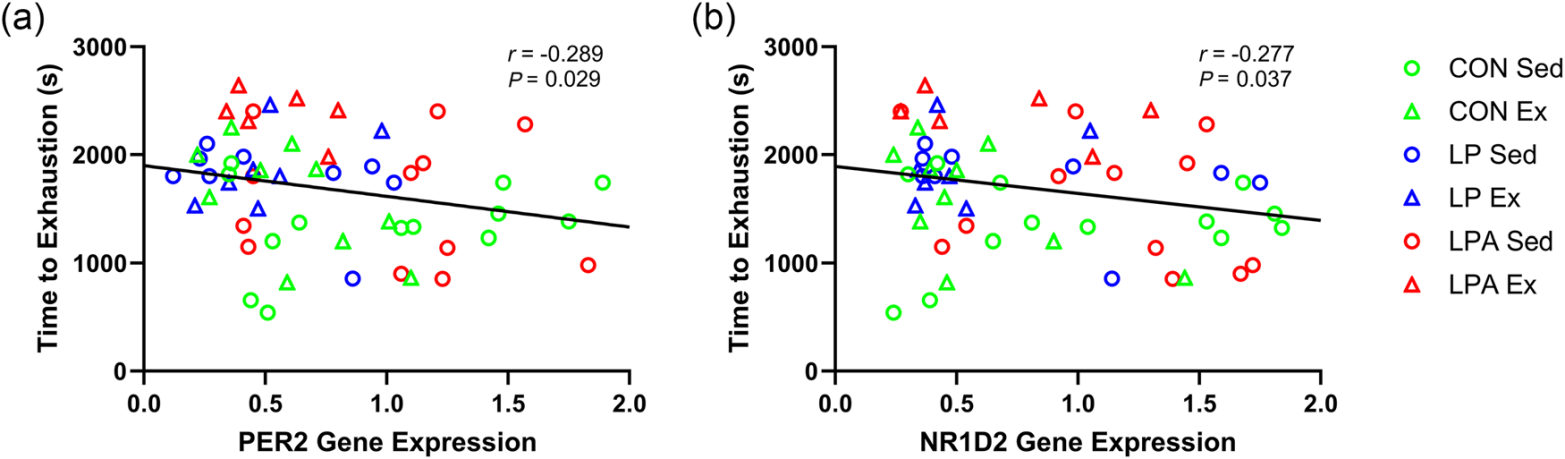
Skeletal muscle soleus correlation examining the relationship of time to exhaustion with individual gene expression of female rats. Relationship of time to exhaustion with *PER2* (a) and *NR1D2* (b). CON Sed, control + sedentary group (*n* = 13); CON Ex, control + exercise group (*n* = 10); LP Sed, probiotic + sedentary group (*n* = 9); LP Ex, probiotic + exercise group (*n* = 7); LPA Sed, Ang(1–7)‐expressing probiotic + sedentary group (*n* = 12); LPA Ex, Ang(1–7)‐expressing probiotic + exercise group (*n* = 6).

There was a weak negative correlation for both *PER2* expression (Figure 14a) and *NR1D2* expression (Figure 14b) with time to exhaustion.

## 4 DISCUSSION

The purpose of this study was to determine the difference in expression of genes related to the circadian clock mechanism in male and female aged rats’ type I (soleus) and type II (tibialis anterior) fibre dominant muscles in response to the administration of an Ang(1–7)‐expressing GMP in conjunction with endurance exercise training over the course of 12 weeks. We observed differentially expressed genes related to the circadian clock at ZT2 (2 h after lights on) within male and female aged rats and also within the soleus and tibialis anterior muscles. Secondarily, we also observed correlations between genetic expression intensity of circadian rhythm genes and markers of performance, time to exhaustion and relative grip strength. Again, these correlations were differentially reported within males and females and within muscles. Therefore, these results indicate our Ang(1–7)‐expressing GMP may exhibit a beneficial additive effect to exercise training by modulating the circadian clock profiles in muscle whilst exhibiting a subsequent potential relationship to performance measures in aged rats, uniquely distinguished between males and females.

Studies have found that the output of the circadian clock declines with age and may contribute to the age‐related decline in tissue function, such as in skeletal muscle (Hood & Amir, 2017; Nakamura et al., 2015; Vitale et al., 2019). The results of this study provide interesting insights in relation to circadian clock gene expression in skeletal muscle and performance measures. We found that our Ang(1–7)‐expressing GMP in combination with endurance exercise training significantly altered the circadian clock and clock output genes differentially in males and females. It can be hypothesised that the changes observed in gene expression at one time point, ZT2, may reflect shifts in the phase of the core clock mechanism in response to the administration of the Ang(1–7)‐expressing probiotic in conjunction with exercise training. However, this hypothesis should be taken with caution as only one time point may not accurately depict phase shifts, but rather an alteration in the amplitude of circadian oscillations only, and should be confirmed with sampling at multiple time points. Alternatively, our study may highlight potential circadian rhythm responses to a zeitgeber introduced at an unaccustomed time (i.e., during the rest phase).

The male tibialis anterior tissue exhibited a decrease in expression of genes that peak at the end of the light/rest phase, including *NR1D1/2*, *DBP*, *CRY2* and *PER1/2*, with a concomitant increase in expression of genes that peak at the end of the dark/active phase, *NFIL3* and *CRY1*. However, these results were not observed within the male soleus muscle nor within either female muscle. The male soleus muscle displayed increased expression of *PER1/2*, *CRY1/2*, *NR1D1/2* and *NFIL3*, indicating a muscle‐specific response to the interventions and potentially reflecting a change in baseline or amplitude. The female muscles displayed little to no change in expression within the remaining target circadian rhythm genes. This suggests that the endogenous clock factor expression of female rats is less responsive to the exercise and Ang(1–7) oral treatments.

Our study displayed sex‐dependent differences in circadian gene expression following exercise and Ang(1–7)‐expressing GMP administration. The association between sex and circadian rhythms has not been fully elucidated, but a recent study has revealed a potential higher rhythmicity in women within the liver and adrenal glands; however, the rhythmicity in skeletal muscle and adipose tissue was unaffected by sex differences (Talamanca et al., 2023). Previous literature suggests men and women exhibit different chronotypes, with women generally having an earlier chronotype (Cain et al., 2010; Duffy et al., 2011; Fischer et al., 2017). However, these differences in chronotype may decrease or even reverse with age (Randler & Engelke, 2019) and may be minimally impacted in our study. Alternatively, circadian rhythm responses to interventions differ between sexes. For instance, caloric restriction has been found to differentially express genes related to circadian rhythm (Astafev et al., 2017). Briefly, sex may affect the genetic expression of *CRY1/2*, *NR1D1* and *RORγ* in the liver in response to 30% caloric restriction, likely playing a role in prolonging the lifespan typically seen in these models. However, it is unclear why sex differences exist concerning the circadian rhythms due to the exclusion of females historically from circadian rhythm research, and it is a current focus of the National Institutes of Health (Beery & Zucker, 2011; Lee et al., 2021; Zucker et al., 2022).

Skeletal muscle processes, such as myogenesis and metabolism, are regulated by an intrinsic circadian clock (Andrews et al., 2010; Dyar et al., 2014) and have been shown to contribute to sleep quality (Ehlen et al., 2017). Alterations in circadian rhythms are associated with decreased physical activity and increased body weight (Qian et al., 2019). Additionally, time‐of‐day and alterations in the circadian rhythm affect muscle performance (Andrews et al., 2010; Douglas et al., 2021). Exercise robustly phase shifts the peripheral circadian rhythms independent of the suprachiasmatic nucleus central clock (Wolff & Esser, 2012). In sedentary adults, chronotype‐dependent phase shifts were reported as a result of morning or evening exercise (Thomas et al., 2020). Morning and evening exercise positively phase‐shifted the circadian rhythms in late chronotypes, whilst evening exercise negatively impacted and early exercise positively impacted early chronotypes. As previously mentioned, women typically have an early chronotype compared to men whilst ageing also shifts individuals to primarily an early chronotype (Cain et al., 2010; Duffy et al., 2011; Fischer et al., 2017). It is possible for the female rats in our study to have an early chronotype compared to the males. This difference in chronotypes could have impacted the differences in circadian gene expression seen within our study. To our knowledge, the chronotypes between sexes in Fisher 344 × Brown Norway rats have not been established.

The current study utilised the tibialis anterior muscle and soleus muscle from both male and female rats. The soleus is primarily composed of type I slow, oxidative fibres, whilst the tibialis anterior is primarily composed of type II fast, glycolytic fibres. Slow‐twitch fibres support long, endurance‐type exercises and fast‐twitch fibres support short, explosive and strength‐type exercises. The slow‐twitch prominent and more chronically recruited soleus muscle has nearly double the number of expressed rhythmic genes compared to the fast‐twitch prominent tibialis anterior muscle in mice (Dyar et al., 2015). Therefore, type I fibre‐prominent muscles display greater flexibility with rhythmic transcription compared to type II fibre‐prominent muscles. Alternatively, recent evidence shows genes related to circadian rhythms do not influence muscle fibre type distribution and downstream circadian rhythms genes are rescued in response to exercise in *BMAL1* knockout models (Dyar et al., 2015; Viggars et al., 2024). In our study, male rat tibialis anterior and soleus tissue displayed differential genetic expression in response to the administration of the Ang(1–7)‐expressing GMP in combination with exercise for *PER1*, *PER2*, *CRY2*, *NR1D1* and *NR1D2*. However, these differences within tissues were not observed in the female rats. Again, this could be due to potential differences in chronotype between sexes. The difference in expression in response to the intervention between tissues in the male rats may be due in part to the fibre type‐specific activities (i.e., the soleus performs primarily as a postural muscle, whilst the tibialis anterior is a performance‐heavy muscle), the timing of these activities for maximal training outcomes, and the overall recruitment patterns of each muscle during endurance exercise. For instance, strength peaks in the afternoon (16.00–20.00 h), with strength training in the afternoons resulting in improved strength and hypertrophy compared to training occurring in the mornings (Douglas et al., 2021; Küüsmaa et al., 2016). However, training daily over an extended period of time close to the time of testing for the type of exercise seems to be more important (Bruggisser et al., 2023). In relation to fibre type‐specific activities, the male soleus may have enhanced the amplitude due to the prominence of slow‐twitch fibres in the muscle in response to the endurance exercise training, likely as a result of the flexibility of rhythmic transcription of type I fibre‐prominent muscles, with the opposite effect occurring in the fast‐twitch‐dominant tibialis anterior muscle. Additionally, the activation and amplitude, as indicated via EMG, of the soleus muscle decrease with an increase in speed, whilst the tibialis anterior activation and amplitude pattern remains relatively stable in rats (Roy et al., 1991). However, the soleus muscle activation patterns are near max at the beginning of exercise and decrease with the increase in speed, likely contributing to the results of this study. Future studies should focus on these sex, fibre type and muscle recruitment circadian rhythms differences.

Of particular interest for this study is the modulation of the RAS using an Ang(1–7)‐expressing GMP to potentially increase the beneficial effects of exercise in an aged rat model. Little is known about the effects of prebiotics and probiotics on circadian rhythms, but the evidence looks promising. The gut microbiome composition influences host homeostasis and is altered by diet and exercise, with dysbiosis linked to disease and obesity (Rijo‐Ferreira & Takahashi, 2019). Host and microbiome circadian rhythms are influenced bidirectionally, as such, with host circadian rhythm dysfunction disrupting microbiome rhythmicity and vice versa (Thaiss et al., 2016; Thursby & Juge, 2017). For instance, *NFIL3* amplitude is influenced by the health of the microbiome, displayed by a reduction in *NFIL3* expression in germ‐free mice (Wang et al., 2017). Additionally, the amplitude of *PER2* and *BMAL1* is increased following the production of 3‐(4‐hydroxyphenyl) propionic acid and 3‐phenylpropionic acid in response to food intake (Ku et al., 2020). A healthy host microbiome may prevent fluctuations of circadian rhythms in response to changes in environmental conditions (Zhang et al., 2023). Alternatively, prebiotics, which help to fuel healthy bacteria in the gut and can alter microbiome composition, have recently been found to reset circadian rhythms following circadian disruptions (Thompson et al., 2021). Due to the influence of the microbiome on host circadian rhythm and recent positive evidence of prebiotics on circadian rhythms, the use of prebiotics and probiotics as an intervention to restore altered circadian rhythms in at‐risk populations should be further elucidated.

Ang(1–7) may directly impact circadian rhythms. For instance, the activation of the Ang(1–7) receptor blunts the increase in heart rate and blood pressure variability typically seen at nighttime with a reduction in baroreflex activation and fluctuations (Wessel et al., 2007). However, this is the only example of Ang(1–7) having a direct influence on circadian rhythms. Our Ang(1–7)‐expressing GMP in combination with exercise training provides novel results on the impact it may have on genetic circadian rhythm targets and warrants further investigation.

Performance may be directly impacted by circadian rhythms, with body temperature having the greatest impact on performance measures (Starkie et al., 1999). Temperature peaks in the early evenings in humans with the concomitant increase in carbohydrate utilization and enhancement of actin–myosin crossbridge cycling, potentially related to peak performance observed at this time point and linking circadian rhythm oscillations (Ranatunga, 2018). Alternatively, *CRY1/2* have been found to repress *PPARδ*, which is responsible for optimising the utilization of lipids as fuel during exercise and may directly impact exercise performance (Jordan et al., 2017).

In our study, we found correlations between our one‐time‐point analysis of gene expression of single targets with time to exhaustion in male and female rats and with relative grip strength in male rats only. *BMAL1* mRNA peaks at the end of the dark/active phase. *BMAL1* expression was positively correlated with time to exhaustion in male soleus and female tibialis anterior tissues, in addition to relative grip strength in male tibialis anterior and male soleus tissues in our study. In support of this, *NPAS2* mRNA expression, which is similar to *BMAL1*, was also positively correlated with time to exhaustion and relative grip strength, but this was only detected in the male tibialis anterior. Lastly, *NFIL3* mRNA, which is also expressed similarly to *BMAL1*, was positively correlated with time to exhaustion in the tibialis anterior muscle in females only. Alternatively, *DBP* mRNA, which peaks antiphase to *BMAL1* (i.e., the end of the light/rest phase), was negatively correlated with time to exhaustion and relative grip strength in both muscles for the males. This pattern was also seen for *PER1* in male tibialis anterior tissue for relative grip strength, *PER2* in male tibialis anterior for relative grip strength and female soleus tissue for time to exhaustion, *CRY1* in male soleus tissue for relative grip strength, and *NR1D2* in female soleus tissue for time to exhaustion. Although there is some variation amongst the genes and muscles in both sexes, these outcomes are consistent with the concept that exercise performance is associated with circadian clock function. This has been demonstrated in mice with significant time‐of‐day differences in endurance and running performance, and these differences are dependent on an intact circadian clock system (Ezagouri et al., 2019). Our correlative results would suggest that this relationship exists in older rats, in particular males. However, the observance of positive correlations of the genetic expression of genes primarily in the early rest phase may be due to the timing of the intervention itself, with oral gavage and exercise training occurring during the early rest phase, potentially exhibiting a training effect on optimal performance measures.

Although the findings presented in this report are novel and important, limitations do exist. Firstly, the absence of young rodents from the study is a major limitation. The addition of young rodents would provide stronger translational insight in relation to ageing and circadian rhythms, whilst also providing meaningful insight into the impact exercise and a GMP may have on circadian rhythms in muscle between young and aged rodents. Secondly, we were unable to determine circadian rhythm oscillations over the course of 24 h. Although this is important in circadian rhythm research, the parameters of our project hindered our ability to perform these measurements at multiple time points. It is important to note that the data presented herein are based on preliminary transcriptomics data reported by Baptista et al. (2023). Thirdly, the small sample size and subsequent unavailability of tissue may have limited statistical power. The COVID‐19 pandemic presented challenges related to tissue collection due to constraints involved with social distancing. There was a discrepancy between groups on the number of available tissues for analyses, with smaller groups potentially impacted the most. Additionally, though not significant, overall survival in male rats (76.8%) was lower compared to female rats (85.7%). This may be due to male rats having a lower life expectancy compared to female rats. It appears unlikely that this difference influenced study outcomes, though the possibility cannot be ruled out. Fourthly, the rats were exercised and gavaged during their rest phase, likely directly impacting our study results. It is possible that the exercise and probiotic intervention during the light phase induced phase shifts or altered the amplitude of circadian gene expressions. Furthermore, our results may provide valuable insight into how skeletal muscle circadian rhythms respond to forced activity during the rest phase, particularly in aged skeletal muscle. Future studies should have the interventions occur during the test subjects’ active phase to reduce any unintended outcomes and also further explore the differential effects of exercise timing on circadian rhythms. Lastly, we only used qPCR analyses to examine genetic expression differences between groups with no follow‐up protein expression analyses. This is due to the unavailability of reliable commercial antibodies for circadian rhythm targets. However, the study design, randomization protocol, tissue collection and data processing and analyses strengthen our study and limit any confounding factors and misinterpretation of the results due to the exclusion of outliers and the appropriate use of statistical analyses.

### 4.1 Conclusions

Herein, we present a novel GMP expressing Ang(1–7) in combination with exercise as a therapeutic approach to potentially alter circadian rhythms in aged rats and display associations between circadian rhythms and physical function. Exercise alone is a prominent external stimulus for altering the circadian rhythm. We display the ability of our GMP to enhance these alterations in circadian genetic expression, particularly in male aged rats. Additionally, expression of certain genes may impact performance measures. A mechanistic approach should be conducted to further elucidate the impact an Ang(1–7)‐expressing GMP may have on altering circadian rhythms.

## AUTHOR CONTRIBUTIONS

All the experiments were conducted in the laboratory at the Division of Gerontology, Geriatrics, and Palliative Care, University of Alabama at Birmingham. Conceptualised and managed the study: Thomas W. Buford. Performed experiments, analysed the data and wrote the manuscript: Emily L. Zumbro, Liliana C. Baptista, Taylor Taylor, Abbi R. Hernandez, Anisha Banerjee, Yi Sun, YouFeng Yang, Qiuhong Li and Thomas W. Buford. All authors have read and approved the final version of this manuscript and agree to be accountable for all aspects of the work in ensuring that questions related to the accuracy or integrity of any part of the work are appropriately investigated and resolved. All persons designated as authors qualify for authorship, and all those who qualify for authorship are listed.

## CONFLICT OF INTEREST

None declared.

## Supporting information



Supplemental Table 1. Time to exhaustion data previously published in Figure 1 of Hernandez *et al.* (2022).

## Data Availability

The data that support the findings of this study are available by reasonable request to the corresponding author.
