# Supplementary material for: Impacts of exercise, renin–angiotensin system modulation or both on skeletal muscle circadian gene expression
Source: Exp Physiol. 2026 Jan 11;111(4):2086–105. doi: 10.1113/EP092318 (PMC13140666; doi:10.1113/EP092318)
Supplement: Supplementary file 1 — Supplemental Table 1. Time to exhaustion data previously published in Figure 1 of Hernandez et al. (2022). [file EPH-111-2086-s001.docx]

**Supplemental Table 1.** Time to exhaustion data previously published in Figure 1 of Hernandez *et al.*, 2022.

|  | **Male** | **Female** |
| --- | --- | --- |
| **Group** | **Mean ± SD** | **Mean ± SD** |
| CON Sed | 507.82 ± 249.11 | 1361.31 ± 411.90 |
| CON Ex | 1388.33 ± 508.15 | 1639.73 ± 505.92 |
| LP Sed | 484.27 ± 270.74 | 1773.22 ± 362.02 |
| LP Ex | 1323.43 ± 486.58 | 1846.27 ± 540.29 |
| LPA Sed | 560.75 ± 241.40 | 1583.42 ± 591.28 |
| LPA Ex | 1329.00 ± 270.43 | 2288.33 ± 505.15 |

CON Sed, control + sedentary group (n = 11, male; n = 13, female); CON Ex, control + exercise group (n = 12, male; n = 11, female); LP Sed, probiotic + sedentary group (n = 11, male; n = 9, female); LP Ex, probiotic + exercise group (n = 7, male; n = 11, female); LPA Sed, Ang(1-7)-expressing probiotic + sedentary group (n = 8, male; n = 12, female); LPA Ex, Ang(1-7)-expressing probiotic + exercise group (n = 6, male; n = 12, female).
